# A Short History of Skin Grafting in Burns: From the Gold Standard of Autologous Skin Grafting to the Possibilities of Allogeneic Skin Grafting with Immunomodulatory Approaches

**DOI:** 10.3390/medicina57030225

**Published:** 2021-03-02

**Authors:** Frederik Schlottmann, Vesna Bucan, Peter M. Vogt, Nicco Krezdorn

**Affiliations:** Department of Plastic, Aesthetic, Hand- and Reconstructive Surgery, Hannover Medical School, Carl-Neuberg-Strasse 1, 30625 Hannover, Germany; bucan.vesna@mh-hannover.de (V.B.); vogt.peter@mh-hannover.de (P.M.V.); krezdorn.nicco@mh-hannover.de (N.K.)

**Keywords:** burn care, allotransplantation, skin transplantation, skin graft, skin substitute, immuno-compatible skin grafts

## Abstract

Due to groundbreaking and pioneering developments in the last century, significant improvements in the care of burn patients have been achieved. In addition to the still valid therapeutic standard of autologous split-thickness skin grafting, various commercially available skin substitutes are currently available. Significant progress in the field of tissue engineering has led to the development of promising therapeutic approaches. However, scientific advances in the field of allografting and transplant immunology are of great importance. The achievement of various milestones over the past decades has provided thought-provoking impulses in the field of skin allotransplantation. Thus, biologically viable skin allotransplantation is still not a part of the clinical routine. The purpose of this article is to review the achievements in burn surgery with regards to skin allotransplantation in recent years.

## 1. Introduction

According to recent surveys by the German Society for Burn Medicine, a total of 4350 people were affected by burn injuries in Germany in 2016 [1]. In Germany, burn patients, especially those with severe burns, are treated in highly specialized burn centers that have comprehensive technical equipment and specialized personnel and can thus ensure optimal care [2]. In initial care, the focus is on immediate treatment and, in particular, adequate volume replacement therapy within the first 24 h, sufficient ventilation, and timely surgical treatment of burn wounds [3]. Due to the enormous progress in the intensive medical care of severely burned patients and the expansion of the network of burn centers, a significant increase in survival rates has been recorded [4,5]. In addition to the above-mentioned intensive care treatment, the interdisciplinary and multimodal treatment concept of burn injuries primarily includes surgical debridement to establish aseptic wound conditions in order to allow sufficient defect coverage and thus adequate wound healing [6,7,8]. Despite intensive research efforts, autologous skin grafting still represents the surgical gold standard in the treatment of burn injuries, as no skin substitute has yet succeeded in sufficiently replacing the function of the patient’s original skin [9]. The gold standard in burn surgery is still autologous tissue transfer, such as autologous split-thickness skin grafting. Skin grafting has developed significantly during the past decades. Thus, in addition to the optimization of autologous skin grafting, the use of allogeneic skin grafts in particular was investigated, but showed limited success. The purpose of this review is to provide an overview of the historical development of allogeneic skin grafting. In addition to the developments of allogeneic skin grafting, the treatment concepts of autologous skin grafting, the status of allogeneic and xenogeneic skin grafts, and the use of synthetic skin substitutes are discussed. Finally, forward-looking perspectives of immunomodulation of allogeneic skin grafts are highlighted, offering a promising approach for future research projects and clinical applications.

### 1.1. Concepts of Autologous Split-Thickness Skin Grafting as the Gold Standard in Burn Surgery

Skin grafting has been practiced by European surgeons for little more than 200 years and can be considered as a standardized procedure nowadays [10]. The history of autologous skin grafting dates back to the first experiments of Giuseppe Baronio in 1804. He investigated skin transplantations in a ram by excising a piece of skin from the dorsum and grafting it immediately on the opposite side. Five out of six skin grafts grew in without problems. In another experiment, Baronio performed transplantation of skin pieces between a cow and a horse. However, without knowing the basic principles of immunology and graft rejection, these attempts of xenogeneic transplantations were not crowned with success [11]. Over the following years, other surgeons such as Johann Friedrich Dieffenbach and Alfred Armand Velpeau focused their research on autologous skin grafting and tried to reproduce the results of Baronio, but hat limited success. It took until 1869 to perform the first successful autologous skin graft on a human by the Swiss-born surgeon Jacques Louis Reverdin [12]. In the following decades, developments and discoveries in the field of allogeneic skin grafting increased rapidly so thatsplit-thickness skin grafting is now clinically well established after many years of experience [13]. Despite intensive scientific efforts, autologous skin grafting, particularly split-thickness skin grafting, represents the workhorse for the reconstructive surgeon in the management of burn injuries. However, donor site limitations and morbidity are major constraints to the use of autologous skin grafts especially in patients with extended skin injuries [14]. In addition, any skin grafting is associated with a donor site defect and aesthetic impairment [3,13]. In particular, permanent wound closure with a satisfactory aesthetic result continues to be a challenge in the treatment of extensive burn wounds. Considering socioeconomic costs for the care of chronic wounds as well as the expected increase in costs due to demographic change, a complete and as early as possible wound closure should be aimed for [15,16].

Since the first successful in vitro cultivation of keratinocytes by Rheinwald and Green in 1975, keratinocytes have increasingly become the focus of research as a promising approach for the production of an artificial skin substitute [17,18]. In the course, various research approaches focused in on the transplantation of autologous keratinocytes [19,20]. For example, autologous keratinocytes are currently commercially available as a cell sheet (EpiCel^TM^, (Vericel Corporation, Cambridge, MA, USA)) [21] or as a spray suspension (ReCell^TM^ (Valencia, CA, USA) or the autologous keratinocyte spray suspension of Deutsches Institut für Zell- und Gewebeersatz gGmbH, Berlin, Germany) [22,23] and are part of the clinical care of burn patients. However, the application remains very limited due to high costs of production and a cultivation time of several weeks [24]. In addition, graft healing rates still vary significantly [25,26]. Both delivery forms also lack the dermal skin component, so that sufficient stability of the grafts, especially in handling during production, but also during transplantation, is not given [3]. The effect on scar formation has also not been conclusively investigated.

### 1.2. The History of Allogeneic Transplantations

The history of burn wound care and treatment dates back to ancient Greece and Rome [27]. The desire to replace lost limbs and facial parts is as old as mankind’s history and many attempts have been made. In reality, ancient allotransplantation attempts all failed and the scientific and surgical success of the first allografts did not occur until about 60 years ago [28]. Due to the severe injuries and burns of war victims in World War II, the foundation for the field of modern transplant biology was laid out of necessity. The young biologist Peter B Medawar was assigned to the service of the British surgeon Dr Thomas Gibson to determine if skin allografts could be used to treat war injuries. In the early 1940s, Medawar began to work on the subject of immune tolerance and transplantation. He published his first fundamental work in this field together with Thomas Gibson in 1943. They proved that the rejection of organs derived from unrelated donors is based on immunological principles [10]. In the following years, Medawar focused on the immunity of homologous skin grafts and their immunological tolerance [29,30,31,32]. For example, he first described the immune privilege of the anterior chamber of the eye using homologous skin grafts [29]. For his fundamental and groundbreaking discoveries, Medawar received a Nobel Prize in 1960 and was knighted [28]. For these discoveries, he is considered the founding father of modern transplantation immunology. However, more than six decades after the first experiments of Medawar, the precise mechanisms of skin allograft rejection are still not fully understood and it has not been possible to achieve reliable clinical tolerance of skin allotransplants [33]. Despite this, human mankind still began to aim for solid organ transplantation. Joseph E Murray, John P Merrill and J Hartwell Harrison in Boston (USA) performed the first solid organ transplant in 1954. They transplanted a human kidney donated between identical twins [34,35]. With advancements in the field of immunology, transplantation and regenerative medicine the transplantation of complex composite tissue allotransplantations became possible. Important milestones in this regard are the discovery and development of immunosuppressive drugs after allotransplantation to prevent rejection making it feasible to transplant facial structures, extremities, larynx, tongue, abdominal wall and external ear and scalp [28]. Along with the growing field of clinical experience in allotransplantation, insights into the pathophysiological and immunological background have been made.

Animal studies have shown that skin grafting between unrelated mouse inbred strains can be expected to occur after ten to 13 days and result in a primary rejection reaction [36,37]. In addition to primary rejection, secondary rejection is also known to occur much more rapidly over the six- to eight-day period and has been observed in repeated transplantation of skin to a recipient animal that had previously rejected a skin graft from the same donor animal [36,37]. The clinical diagnosis of rejection is made based on various parameters, such as clinical symptoms in the form of epidermolysis, hemorrhage, or necrosis, as well as laboratory chemical analyses and histologic evaluation of tissue biopsy. In this context, important rejection features include the infiltrating immune cells and their ratio to each other, structural matching of tissue anatomy, and histological confirmation of damage to local blood vessels [38,39,40]. As shown later, the compatibility of major histocompatibility complex (MHC) molecules between donor and recipient can significantly improve the transplantation outcome and prevent rejection [41,42]. In recent years, the success of organ transplantation in particular has been significantly improved by matching the MHC profiles between donor and recipient [42,43]. However, even accurate MHC typing of the graft cannot always prevent rejection of the recipient organism. A perfect match is rarely achievable in clinical practice because the donor and recipient must usually be related to have a genetic similarity of MHC molecules. Moreover, because of a mismatch between the supply and demand of donor organs, the selection of an MHC-compatible organ is severely limited [44]. In humans, the genes encoding MHC molecules are located in the human leukocyte antigen (HLA) gene region and has high genomic diversity [45,46,47]. Due to this, an ideal donor who has an identical genomic MHC profile is usually not available despite an existing relationship [42,44,47]. The reasons for this are, in particular, that despite extensive progress the available methods of HLA typing make accurate typing almost impossible due to polygenetic diversity and the resulting complexity [47]. Moreover, it is known that even MHC-identical transplants, for example in HLA-identical siblings, are rejected with a time delay. The rejection reaction is based on the minor histocompatibility antigens, which are individual-specific antigens that are not part of the MHC proteins [48,49,50]. Minor histocompatibility antigens will not be discussed in detail here.

Despite various research approaches, it has not yet been possible to inhibit the specific immune response against allografted tissue without compromising the immune response of the entire recipient organism [51]. Therefore, most transplantations often require the use of immunosuppressive drugs that cause general immune compromise [51,52]. Thus, clinical success in solid organ transplantation is largely attributable to advances in the development of immunosuppressive drugs. However, systemic immunomodulation of an organism can be toxic and is associated with impaired immunocompetence and thus an increased risk of infection [51]. The main driver of success of allogeneic tissue or organ transplantation depends on MHC typing of the graft, the use of effective immunosuppression, and the operative skills of the surgeon performing the procedure [53,54]. In particular, for the severely burn patient, the immunologic derailment in the setting of systemic inflammatory response syndrome prohibits additional drug immunosuppression [55]. The immunologic rejection reaction of allogeneic skin grafts in severely burned patients has been intensively studied and described. To sum up, the allogeneic skin graft can cause a potent immune response involving both the innate and adaptive immune system of the host’s organism. T cell mediated mechanism as well as B cell and NK cell activation play a significant role in the process of allograft rejection [56]. In conclusion, immunomodulation of allogeneic grafts would be a promising way to reduce rejection. Interference with the intrinsic alloreactivity of the whole organism by means of immunosuppressive drugs could thus be prevented. In particular, the application in immunocompromised patients, such as severely burned patients, would be extremely promising regarding the immuno-compatibility of transplants and would enable a diverse clinical use of, for example, allogeneic skin transplants without rejection.

### 1.3. Current Use of Allogeneic Skin Grafts in the Care of Severely Burned Patients

Despite the above-mentioned disadvantages of allotransplantations, allogeneic skin grafts have a place in the care of severely burned patients to a certain extent and are widely used for wound management in burn centers [57]. Autologous skin grafts are currently commercially available as cryopreserved and glycerol-preserved skin sheets via biobanks or skin banks all over the world [57,58]. The history of skin biobanking dates back until to the 1990s [59]. Transplantation of cryopreserved and glycerol-preserved and thus biologically unviable allogeneic skin from body donors is also clinically indicated in some cases because of the lack of autologous donor sites for split-thickness skin grafting [60]. Functional as biologic dressing, it can not only provide ideal temporary wound coverage in extensive burns when autologous skin grafting is not immediately available but also prepare the wound bed for definitive wound closure [57]. However, the use of allogeneic skin grafts are not promising. In particular, secondary graft rejection due to an immune reaction induced by the graft, as well as secondary infections and increased scarring, are disadvantages in their use as permanent skin substitutes [61]. The antigenic properties of the foreign skin can be reduced by glycerol preservation, resulting in the demise of cellular epidermal and dermal components [62]. Due to the immunocompromised state of the severely burn injured patients, delayed rejection may also occur during the course after the patient’s immunocompetence is restored. The only advantage of using allogeneic material has been shown to be the immediate restoration of skin barrier function in terms of a biological dressing [62]. The aesthetic results after transplantation of allogeneic skin are usually unsatisfactory. Incongruent healing rates of allogeneic skin grafts have been reported in some cases [3]. To sum up, allogeneic skin grafting has its position in the clinical care of severely burned patients, but at the same time goes hand in hand with a plethora of limitations and disadvantages.

### 1.4. Current Use of Xenogeneic Skin Grafts in the Care of Severely Burned Patients

In addition to the gold standard of autologous skin grafting, xenogeneic materials, such as porcine skin and fish skin, are used for temporary defect coverage, particularly in large-scale burns [61,63]. It has been reported elsewhere that the use of porcine skin and fish skin in severely burned patients was associated with a significant reduction in intravenous fluid use, pain scores and pain medication. This allowed sufficient stabilization of the patient until definitive treatment of the skin defects by autologous skin grafting [64,65]. Other reports suggest, that the Nile tilapia skin (Orechromis niloticus) has noninfectious microbiota, a morphological structure similar to that of human skin, and therefore showed good outcomes when used as a xenograft for treatment of experimental burns [66]. A phase II randomized controlled trial even described complete re-epithelialization in burn wounds without autologous split-thickness skin grafting [67]. The research results in this regard appear promising, but further studies are needed to evaluate the potential use compared to autologous skin grafting as current gold standard in clinical routine. In conclusion, xenogeneic skin grafts have a place in the clinical care of severely burned patients, but at this time can only be considered as a temporary solution.

### 1.5. Skin Substitutes as an Alternative to Autologous Skin Transplantation

Over the years, a wide range of industrially manufactured skin substitutes have been established more or less successfully, so that a potpourri of purchasable skin substitutes are currently available on the market [3,68]. Each of these skin substitute materials plays a part in various research approaches and, in some cases, is also used in the clinical care of burn patients [69,70].

Dermal skin substitutes can be broadly categorized as decellularized dermis derived from human or animal sources, artificially constructed scaffolds comprised of highly purified biomaterials or entirely synthetic polymers. In view of the fact that there are dozens of different commercially available skin substitute matrices on the market, only MatriDerm^®^ (MedSkin Solutions Dr. Suwelack AG, Billerbeck, Germany) and NovoSorb^®^ Biodegredable Temporising Matrix (BTM) (PolyMedics Innovations, Denkendorf, Germany) will be presented here as examples in detail, since they are currently standard in clinical use and show promising results. However, Table 1 provides an overview of other dermal skin substitutes of biological and synthetic origin currently available on the market.

MatriDerm^®^, a dermal, cell-free skin substitute for deep-seated burns, has been commercially available for several years. The matrix consists of bovine collagen type I, collagen type III and elastin. As studied in animal models, MatriDerm^®^ matrix is converted into endogenous matrix by the recipient organism over a period of weeks [71,77]. Similar biodegradable behavior has also been demonstrated in the human organism [78]. MatriDerm^®^ is available in various thicknesses and can be stored at room temperature, making handling considerably easier than with other biomaterials [71]. In particular, the single-stage procedure with simultaneous autologous split-thickness skin grafting is clinically considered a significant advantage and showed no inferiority to allogeneic split-thickness skin grafting alone [3]. Better scar quality as well as reduced wound contraction were observed [79,80], so the results to date in clinical application are promising [80,81]. However, full-thickness skin replacement with MatriDerm^®^ is so far only possible in combination with autologous split-thickness skin grafting.

NovoSorb^®^ BTM is a fully synthetic dermal skin substitute that eliminates any risks of cross-species residual antigenicity. It consists of biodegradable polyurethane foam with a temporary non-biodegradable polyurethane seal. Thereby, it is easy and inexpensive to produce [76]. A further advantage is the avoidance of cross-species immune rejection or disease transmission as well as circumventing ethical and cultural objections to the use of animal derived products. A plethora of proof-of-concept studies using NovoSorb^®^ BTM has been conducted to determine its safety and ability to provide permanent wound closure when combined with split-thickness skin grafts in a two-stage procedure in sheep, pigs and humans [82,83]. Furthermore, the particular abundance of host inflammatory cells in athymic nude mice receiving full-thickness skin excision followed by grafting of the dermal NovoSorb^®^ BTM template, showed evidence that new collagen deposition and neovascularization with BTM had a more extensive vascular network compared to Integra^®^ (Integra LifeSciences, Plainsboro, NJ, USA) [84]. NovoSorb^®^ BTM demonstrates favorable properties as dermal skin substitute but further studies are needed to evaluate its position in clinical routine, especially for the treatment of burns.

### 1.6. Tissue Engineering for Skin Reconstruction

Tissue engineering of biologic skin substitutes has developed over the last few decades from individual applications of skin cells or biologic scaffolds to the combination of cells and scaffolds for treatment and healing of chronic skin wounds and burns. Several tissue engineered skin substitutes have overcome the disadvantages of autologous split-thickness skin grafting as well as the immunogenicity of skin allotransplantation. Most research approaches focus on the use of keratinocytes as a cell pool for tissue engineering approaches [20]. Autologous keratinocytes may persist indefinitely and provide permanent wound closure whereas allogeneic keratinocytes will only remain in the wound bed for days to weeks [85,86]. However, there are still remaining disadvantages such as high production costs, time consuming processes and culture conditions for each patient regarding the application of autologous keratinocytes [87]. Recently, major progress has been made in this field so that cultured autologous keratinocytes as a source for bioengineering can be produced in unlimited supplies by establishing cost-effective culture protocols and increasing their proliferative capacity [88,89]. Allogeneic keratinocytes can be provided off-the-shelf with less manufacturing challenges, but immune compatibility is a major concern with their use as mentioned above.

Despite single cellular tissue engineering approaches, combinations of keratinocytes and dermal skin substitutes have been investigated intensively to produce a skin substitute that is comparable to human healthy skin. For example, MatriDerm^®^ was used as a matrix for cell-based studies to investigate its applicability as a carrier matrix for tissue engineering approaches due to its promising properties and effects on the human organism. Cells of various origins, such as pancreatic stem cells [90], preadipocytes [91], fibroblasts, and keratinocytes [92,93], have been shown to adhere and proliferate on the MatriDerm^®^ matrix in vitro. This makes MatriDerm^®^ suitable as a carrier matrix for further research approaches in the field of tissue engineering and regenerative medicine [92]. Since 2006, advanced models of tissue engineered skin substitutes have been described but all showed limited success [94,95,96]. With the growing field of skin tissue engineering, regulatory environments and requirements have expanded. Among the several determination of safety and efficacy, the determination of medical risks is a fundamental factor limiting the clinical applicability of novel tissue engineered skin constructs [97].

Despite the described disadvantages, allogeneic keratinocytes represent an important cell therapeutic tool, which could be further optimized by immunomodulation. With the establishment of viral vectors as a transfection method, efficient transfection of keratinocytes was achieved [98,99,100]. In this regard, viral transfection of keratinocytes represents a promising tool for gene therapy applications. For example, Vogt et al. demonstrated expression of growth factors and ß-galactosidase and regeneration of the epidermis after transplantation of retrovirally transfected keratinocytes in a porcine model [101]. Systemic expression of genes in bioreactor systems also offers multiple options with respect to gene therapy application of modified keratinocytes [102]. However, a careful risk-benefit analysis should be performed when using viral vectors. On the one hand, they can induce an uncontrolled immune response and, on the other hand, they only allow the transfer of genes of a limited size [103,104]. Despite intensive research and efforts, data on the application of genetically modified keratinocytes as skin substitutes in vivo remain insufficient. In conclusion, genetic modification of keratinocytes could provide the basis for the production of a universally applicable and immunologically compatible skin substitute [105].

Another potential approach for skin tissue engineering is the use of three-dimensional (3D) bioprinting. Bioprinting, involving computer-controlled deposition of scaffolds and cells in a plethora of shapes and patterns, can offer the potential to fully replicate native human skin [106]. As described in literature, multilayered approaches using fibroblast, keratinocytes as well as collagen have been made to bioprint human skin [107,108]. In comparison with traditional methods for skin engineering, 3D bioprinting offers several advantages in terms of shape- and form retention, flexibility, reproducibility, and high culture throughput. Therefore 3D bioprinting is a promising technology that can achieve rapid and reliable production of cellular skin substitutes, satisfying both clinical and industrial need [106,109]. Although 3D bioprinting shows promising results so far, the research is still in its infancy and further studies are needed to overcome the disadvantages such as vascularity, optimal cell and scaffold combinations and production costs [106,109].

### 1.7. Future Perspectives: Universal Off-the-Shelf Transplantable Skin Substitutes with Low Immunogenicity

Despite the wide range of skin substitutes available, autologous split-thickness skin grafting is still usually required for complete and sufficient wound closure. Although the ultimate treatment goal in plastic surgery is replacing “like with like”, donor site tissue is often a limited source. Autologous skin grafts as above-mentioned have the advantages of living tissue allografts that derive from the patient itself and are not rejected. One of the most promising research approaches for future generations of patient-specific skin substitutes is the use of immunologically or genetically modified autologous keratinocytes due to their almost unlimited availability as they can overcome graft rejection and immune incompatibility. Morgan et al. first described the successful in vitro gene modification of keratinocytes by applying the method of retroviral gene transfer. They transferred a recombinant human growth hormone gene into cultured human keratinocytes, transplanted them into athymic mice and observed epithelial regeneration [110]. Especially during recent years, many different approaches have been made to generate a universal off-the-shelf transplantable skin substitute for cosmetic and reconstructive surgery [111]. However, gene therapy for the skin has been investigated intensively but showed limited success overall [112]. Several studies have demonstrated that modulation of MHC-I expression could significantly influence survival and acceptance of transplanted allogeneic organs. In a knockout mouse model lacking MHC-I and MHC-II expression, allogeneic skin grafts showed delayed rejection kinetics [113]. Auchincloss et al. demonstrated that MHC-II-deficient skin grafts exhibited graft rejection without delay in the mouse model [114]. The importance of MHC-I expression in allogeneic organ rejection was then investigated in further studies that focused on suppressing antigen presentation directly to the allograft. To achieve this, β2-microglobulin (β2M) and transporter-associated-with-antigen-processing (TAP)-deficient transplantation models were developed and further investigated. It was shown that in β2M-deficient mice, pancreatic islet allografts exhibited unlimited graft survival [115], whereas heart and liver allografts showed only delayed graft rejection [116]. Allografted kidneys showed significantly improved renal function of the graft [117]. In a further step, the rejection behavior of skin grafts in TAP- and in β2M-deficient mouse models was also investigated. This demonstrated delayed rejection of the allograft [118,119,120]. Despite intensive efforts, complete removal of the MHC class I molecule in the allograft has not yet been achieved by eliminating the β2-microglobulin or TAP transporter.

Genetic modification of cells to achieve better immuno-compatibility has been investigated in further in vitro studies. For example, using specific intrabodies in endothelial cells, a phenotypic knockout of MHC-I expression was achieved by retention of the molecules in the endoplasmic reticulum [121]. As another approach to allografting keratinocytes and skin, a significantly reduced MHC-I expression was demonstrated in human and monkey cell lines by using specific intrabodies. The cell lines showed different HLA-A, -B, and -C haplotypes [122]. However, due to the high genomic diversity of HLA genes, genotypic knockout of MHC-I molecules has not been feasible to date. To improve the acceptance of allogeneic skin grafts, it is desirable to suppress MHC-I expression and thus prevent rejection. Based on previous studies, the use of viral MHC-I modulatory proteins or vectors represents an innovative and promising research approach to modulate the expression of MHC-I molecules and thus prevent graft rejection. A plethora of experimental methods exists to create universal allogeneic keratinocytes that can overcome host cellular immune response.

In the course of coevolution, many viruses have developed the ability to suppress viral peptide presentation and thus, do not elicit an immune response from the infected organism [123,124]. A prominent example is human cytomegalovirus (HCMV), which, by reducing MHC-I expression on the cell surface of infected cells, does not elicit viral peptide presentation and no immune response from the infected organism [125,126]. Absence or reduction of MHC-I expression prevents activation of CD8+ cytotoxic T lymphocytes [127,128,129]. The underlying molecular mechanisms are well understood so far. For example, Ahn et al. demonstrated that HCMV uses various strategies to avoid being recognized by the immune system of the infected organism [130]. HCMV prevents MHC-I expression at the cell surface through various modulatory transmembrane glycoproteins called unique short glycoproteins (US). In particular, it has been shown that US2, US3, US6, and US11 glycoproteins are responsible for MHC-I modulation [126,131]. Thus, the use of US glycoproteins represents a promising approach to modulate the expression of MHC-I molecules in allografts and to prevent graft rejection. As published earlier by Schlottmann et al. human primary keratinocytes could be transfected using viral US11 vectors, resulting in a temporary reduction of MHC-I surface expression. Furthermore, they seeded transfected keratinocytes on MatriDerm^®^ matrices and observed long-term cell survival as well as histological patterns comparable to healthy human skin [132]. The viral vector used was characterized by greater loading capacity while maintaining moderate immunogenicity and inflammatory components, thus representing a promising research approach. Nevertheless, vector tropism, the duration of transgene expression as well as vector immunogenicity are crucial issues and sometimes even disadvantages that have to be taken into account to allow efficient, specific and safe applications of viral vectors in transgene therapeutic approaches [133].

Gene editing can be another feasible approach for immunogenic modification of allogeneic keratinocytes. The significant advances in the field of gene therapy, especially using the Clustered Regularly Interspaced Short Palindromic Repeats/Cas9 (CRISPR/Cas9) system, could represent another promising research approach to generate an immuno-compatible and universally applicable skin substitute. In this regard, research approaches using viral vectors could be replaced, so that the vector associated risks can be reduced. However, no approach to generate a skin construct using CRISPR/Cas9 has yet been sustainably established in the literature [134,135,136].

Another approach for a hypogenic skin graft engineered with universal cells has been recently described by Deuse et al. [137]. Endothelial cells, smooth muscle cells and cardiomyocytes derived from hypoimmunogenic mouse or human induced pluripotent stem cells showed inactivated MHC class I and II genes and an overexpression of CD47. Further experiments showed reliably immune evasive rejection in fully MHC-mismatched allogeneic recipients and long-term graft survival without the use of immunosuppressive medication [137].

However, the use of HLA silenced cells is not without clinical concerns as it remains uncertain as to what level HLA genes should be silenced in vivo or in vitro to evade host CD8+ cytotoxic T cell recognition or natural killer cell (NK cell) activation. Genetically modified allogeneic cells might therefore be invisible for T cell-based immune responses that are essential for the lysis of infected or oncogenically transformed cells as well as apoptosis. In addition, the results of Christinck et al. and Sykulev et al. have shown that 1 to 200 MHC-I molecules per cell can be sufficient to induce an immune response in the recipient organism after transplantation [138,139]. Therefore, there is a considerable need for further studies that investigate the immunological responses of allogeneic modified keratinocytes in vitro and in vivo.

## 2. Conclusions

Mankind’s desire to replace whole organs and tissues with allogeneic transplants goes back over centuries. Significant successes, especially in burn surgery and consequently allogeneic skin transplantation for treatment of extensive burn wounds, could only be recorded in the last decades. As described in the preceding sections, despite intensive efforts, it has not yet been possible to produce a skin substitute that forms a full-fledged, universally applicable and immunologically compatible approach and whose application allows an appealing aesthetic result [140,141,142,143,144]. The first autologous skin graft was described in 1804. Since then, the autologous skin grafting procedure has been intensively researched and further developed, so that autologous split thickness skin grafting is nowadays the gold standard in the treatment of severely burn injured patients. However, as summarized in this review, allogeneic and xenogeneic skin grafts also have a place in the clinical care of severely burned patients. The first discoveries of transplantation immunology by Medawar et al. have led to far-reaching developments in the field of transplantation surgery, so that nowadays solid organs can finally be transplanted as well. However, allogeneic transplants still carry the risk of rejection, so that intervention in the intrinsic alloreactivity of the recipient organism, for example by immunosuppressive drugs, is necessary. To address these issues, a number of commercially available skin substitute matrices have been developed by the industry. In this review, MatriDerm^®^ and NovoSorb^®^ BTM were presented as examples of dermal skin substitute matrices. However, no commercially available skin substitute has achieved the properties of healthy human skin. An ideal skin substitute would have to ensure, if possible, an early restoration of the physiological and anatomical function of the skin. Currently, this ideal can only be provided by the use of full-thickness skin grafting or flap plasty [96]. An ideal skin substitute should sufficiently restore the function of the skin as a physical and chemical barrier against external influences [145]. In addition, it requires sufficient biomechanical stability as well as high biological functionality in order to allow sufficient loading capacity and to prevent a rejection reaction [146,147]. The process of thermoregulation should also be enabled by sufficient adhesion to the wound bed, sweating, and water diffusion [96,148]. Degradation of the skin substitute and remodeling into autologous tissue would also have to be present. The above-mentioned characteristics depend largely on the chosen skin substitute procedure. The biomaterials used require a basic framework that has neither antigenic nor toxic properties and can also ensure directed cell migration and proliferation [149,150]. Easy handling in application, rapid and inexpensive availability, and aesthetic results also play a decisive role in the use of skin substitute materials [148,151]. In addition to the industrial developments, the broad field of skin tissue engineering should not be ignored, which has been able to reveal many promising research approaches. Despite various scientific and industrial achievements during recent years as well as developments in burn care, there is no therapy with a skin substitute that shows promising aesthetic, functional, and immuno-compatible results based on acceptable and sufficient manufacturing. Therefore, forward-looking research approaches reveal immunological modification of allogeneic skin grafts. Thus, an ideal skin substitute with sufficient biomechanical properties would become available with comparable properties to healthy human skin and simultaneous immunological compatibility. The long-term goal remains the generation of an off-the-shelf transplantable skin substitute with high biological functionality and reduced immunogenicity. However, until this goal can be achieved, extensive research efforts are required to generate further results.

## Figures and Tables

**Table 1:** Overview of dermal skin substitutes currently commercially available.

Name of Dermal Skin Substitute	Material	Literature
MatriDerm^®^	bovine collagen type I, collagen type III and elastin	[71]
Dermagraft^®^	neonatal-derived bioengineered tissue comprised of dermal fibroblasts	[72]
AlloDerm^TM^	donated allograft human dermis, processed to remove cells while preserving biologic components and structure of the dermal matrix	[73]
Integra^®^	dermal component of bovine collagen type I and shark chondroitin-6-sulfate directed to the wound side and an outwardly directed silicone membrane	[74]
denovoSkin^TM^	hydrogel from a dermo-epidermal component after cultivation from autologous skin tissue samples	[75]
NovoSorb^®^ BTM	biodegradable polyurethane foam with a temporary non-biodegradable polyurethane seal	[76]
